# The common rejection module in chronic rejection post lung transplantation

**DOI:** 10.1371/journal.pone.0205107

**Published:** 2018-10-05

**Authors:** Annelore Sacreas, Joshua Y. C. Yang, Bart M. Vanaudenaerde, Tara K. Sigdel, Juliane M. Liberto, Izabella Damm, Geert M. Verleden, Robin Vos, Stijn E. Verleden, Minnie M. Sarwal

**Affiliations:** 1 Leuven Lung Transplant Unit, Department of Chronic Diseases, Metabolism, and Ageing (CHROMETA), KU Leuven, Leuven, Belgium; 2 Division of Transplant Surgery, Department of Surgery, University of California, San Francisco, San Francisco, California, United States of America; University of Michigan, UNITED STATES

## Abstract

**Rationale:**

Recent studies suggest that similar injury mechanisms are in place across different solid organ transplants, resulting in the identification of a common rejection module (CRM), consisting of 11 genes that are overexpressed during acute and, to a lesser extent, chronic allograft rejection.

**Objectives:**

We wanted to evaluate the usefulness of the CRM module in identifying acute rejection (AR) and different phenotypes of chronic lung transplant rejection (CLAD), i.e., bronchiolitis obliterans syndrome (BOS) and restrictive allograft syndrome (RAS), using transbronchial brushings, broncho-alveolar lavage (BAL) samples, and explant tissue.

**Methods:**

Gene expression measurements for the 11 CRM genes (CD6, TAP1, CXCL10, CXCL9, INPP5D, ISG20, LCK, NKG7, PSMB9, RUNX3, and BASP1) were performed via qRT-PCR in 14 transbronchial brushings (AR, n = 4; no AR, n = 10), 32 BAL samples (stable, n = 13; AR, n = 8; BOS, n = 9; RAS, n = 10), and 44 tissue specimens (unused donor lungs, n = 15; BOS, n = 13; RAS, n = 16). A geometric mean score was calculated to quantitate overall burden of immune injury and a new computational model was built for the most significant genes in lung transplant injury.

**Results:**

Acute rejection showed a significant difference in almost every gene analysed, validating previous observations from microarray analysis. RAS tissue demonstrated a higher geometric mean score (6.35) compared to donor tissue (4.09, p = 0.018). Analysis of individual CRM genes showed an increased expression of ISG20, CXCL10 and CXCL9 in RAS. In BAL samples, no differences were detected in gene expression or geometric mean scores between the various groups (stable, 5.15; AR, 5.81; BOS, 5.62; RAS, 7.31). A newly modelled 2-gene tissue CRM score did not demonstrate any difference between BOS and RAS (p>0.05). However, the model was able to discriminate RAS from BOS tissue (AUC = 0.75, 95% CI = 0.55–0.94, p = 0.025).

**Conclusion:**

Transcriptional tissue analysis for CRM genes in CLAD can identify acute rejection and distinguish RAS from BOS. The immune activation in RAS seems similar to acute rejection after kidney/liver/heart transplantation.

## Introduction

Chronic transplant rejection remains one of the major complications following solid organ transplantation, leading to graft loss and mortality, with acute rejection being a major risk factor to develop subsequent chronic rejection [1]. Although the mechanisms of rejection remain largely unknown, it is considered to be a chronic humoral and cell-mediated response of the recipient towards the implanted non-self donor organ, leading to irreversible tissue fibrosis, failure of the organ, and eventually graft loss [2]. Therefore, one could assume that similar mechanisms of rejection are in place across different engrafted organs.

This assumption led researchers to combine data from across multiple organs and resulted in a common rejection module (CRM), consisting of 11 genes (CD6, TAP1, CXCL10, CXCL9, INPP5D, ISG20, LCK, NKG7, PSMB9, RUNX3, and BASP1) that were overexpressed during allograft rejection. This composite transcriptional score quantified injury and identified patients with an increased risk of future fibrosis [3]. The CRM genes, originally identified upon a meta-analysis of public microarray gene expression data for biopsy-confirmed acute rejection in four different organs (kidney, lung, heart, and liver), was found to quantify inflammation in the tissue in all organ rejection settings, irrespective of tissue source. Subsequently, the clinical utility of these genes was validated by PCR amplification of the signal in an independent set of kidney transplant patient biopsies with acute rejection, whereby the assay could be run at low cost, high throughput and with minimal computation for a 4 hour turn-around time from sampling to results [4].

Amongst all types of solid organ transplants, lung allografts are the most immunogenic, with 40–70% of grafts eventually being rejected [5] and a median time to rejection of 5.7 years post lung transplantation (LTx) [6]. Chronic rejection post-LTx, is believed to occur in at least two pathologically distinct clinical phenotypes, either bronchiolitis obliterans syndrome/BOS or restrictive allograft syndrome/RAS, and both pathologies are combined under the generic umbrella of chronic lung allograft dysfunction (CLAD) [7]. BOS is characterized by persistent obstructive pulmonary function decline, air trapping on expiratory CT scan, and obliterative bronchiolitis on histopathology. RAS is characterized by a restrictive pulmonary function decline, accompanied by persistent pleuroparenchymal infiltrates on CT scan, and patterns of pleuroparenchymal fibro-elastosis on histopathology. Clinically, the distinction between these two forms is important as BOS patients experience a survival of 3–5 years post-diagnosis, while survival for RAS patients is limited to 0.5 to 1.5 years [8]. Thus, evaluating non-invasive assays that can distinguish between these two pathological entities would be important for graft prognosis.

In this study, we evaluated the application and utility of the CRM genes in acute rejection and CLAD post-LTx with special consideration of the two different phenotypes, BOS and RAS. The CRM genes were profiled for acute rejection in a small set of transbronchial brushing samples and for chronic rejection in tissue samples from explanted end-stage CLAD lungs. Moreover, as the CRM score was previously found to be elevated in both kidney tissue and matched urine samples during acute rejection [9], we aimed to determine CRM expression in broncho-alveolar lavage (BAL) as well.

## Material and methods

### Ethics statement

This study was approved by the ethical committee of the University Hospital of Leuven (S58926). Written informed consent to participate in tissue and lavage biobanking (S51577) was provided by all patients included in this study or by next of kin. Unused donor lungs were collected following ethical approval (ML6385, S532174) and under existing Belgian law stating that organs of insufficient quality for transplantation can be used in approved research programs. Given this law, no written informed consents were necessary or obtained from donors or next of kin. Additionally, none of the transplant donors were from a vulnerable population.

### Study cohort

The diagnosis of CLAD was made following international guidelines [10]. Chronic rejection CLAD was diagnosed as a persistent decline in forced expiratory volume in 1 second (FEV_1_) ≥20% in the absence of other identifiable causes. CLAD was further classified as either RAS or BOS: the RAS phenotype was diagnosed using restriction on pulmonary function (either forced vital capacity, FVC, decline ≥20% or total lung capacity, TLC, decline ≥10% compared to the best baseline post-LTx) in combination with the presence of persistent infiltrates on CT scan. In other cases, BOS was diagnosed. Histopathological analysis of the contralateral lung was used to consolidate our phenotype diagnosis (i.e., normal parenchyma in BOS and interstitial fibrosis and pleural thickening in RAS). In the event that patients evolved from one phenotype to another, as previously described [11], the last phenotype prior to re-transplantation or death was used for analysis.

### Transbronchial brushings (TBB)

To evaluate if the gene expression changes in bronchial brushings could accurately reflect changes in the CRM genes, similar to the tissue, and correlate with a histopathological diagnosis of acute rejection, cell samples obtained from bronchial brushings of LTx recipients with (n = 4) and without (n = 10) acute rejection (AR) were used for CRM gene expression measurement. All TBB were obtained under fluoroscopic guidance during bronchoscopy within the first year post-LTx. The cytology brush is passed through the bronchoscope to the desired site where the lesion is brushed and then retracted into a protective sheath to avoid loss of specimen during withdrawal.

### Broncho-alveolar lavage (BAL)

BAL is performed routinely as part of our follow-up at day 1, 21, 90, 180, 360, 540 and 720 post-LTx and additionally when infection or acute/chronic rejection is suspected. Therefore, at CLAD diagnosis, BAL samples were available in a proportion of patients (BOS, n = 9; RAS, n = 10) of which lung tissue was also collected. Additionally, BAL samples of patients with AR (n = 8) were included. As controls, BAL samples of patients at post-operative day 720 without evidence of any disease and who were CLAD-free until at least the 1^st^ January 2017 (n = 13, average of 5.7 years CLAD-free) were used. BAL was performed with 2x50 cc of saline, of which the recovered fractions were pooled following gentle aspiration. BAL was used for differential cell count, microbiology, virology and biobank for research purposes. The cell pellet was used for measurement of CRM gene expression.

### Lung processing

From 2009 onwards, human explant lungs at the time of re-transplantation or autopsy were collected and processed in our research lab, as previously described [12]. In brief, air-inflated lungs were frozen and cores of 1.4 cm diameter and 2 cm length were extracted. For this study, one randomly selected core per lung was used. Tissue samples of 13 BOS lungs and 16 RAS lungs were used. Donor lung samples were selected from regions free of anomalies (reason for decline: persistent embolization, n = 6; infection, n = 3; onset of lung disease, n = 2; contusion, n = 1; kidney tumour, n = 1; rupture of artery, n = 1; unexpected death of recipient, n = 1).

### CRM gene expression measurement

Total RNA was extracted from each tissue core, BAL sample cell pellet and TBB sample using TRIzol Reagent (Invitrogen, CA, USA).RNA integrity was ensured using the RNA 6000 NanoLab Chip Kit (Agilent Technologies, CA, USA)and a 260/280 ratio of at least 1.4 and 1.8 was ensured for BAL and TBB/tissue samples respectively [13–15]. Using SuperScript VILO Master Mix (Invitrogen), as per the manufacturer’s protocol, 25 ng of extracted quality total RNA was reverse transcribed into cDNA. Specific target amplification was performed on 3.125 ng relative amount of cDNA using pooled individual TaqMan real-time assays for the 11 genes: CD6, TAP1, CXCL9, CXCL10, INPP5D, ISG20, LCK, NKG7, PSMB9, RUNX3, BASP1. These were investigated in a multiplex with TaqMan PreAmp Master Mix (Life Technologies, CA, USA) in a thermal cycler (10 μl final volume, 18 cycles, Eppendorf Vapo-Protect, Hamburg, Germany). cDNA was then diluted 1:20 with sterile water (Gibco, Invitrogen). Quantitative real-time PCR was performed on the QuantStudio 6 Flex System (Life Technologies) using 5 μl of the diluted sample from the specific target amplification, along with the TaqMan Gene Expression Master Mix (Life Technologies) under standard conditions (2 min at 50°C, 10 min at 95°C, 40 cycles of 15 s at 95°C, 1 min at 60°C) using TaqMan gene expression assays (Life Technologies, Invitrogen) for each of the 11 genes investigated. The relative amount of mRNA expression in each sample was calculated using the comparative threshold cycle (Ct) method. Ribosomal 18S RNA (18S) and Universal RNA (Qiagen, Venlo, The Netherlands) were used for normalization of all genes since they showed the least variability in gene expression across all samples. Final gene expression results were converted to fold change. A composite CRM score was defined for each individual sample (be it tissue or BAL) by calculating the geometric mean of the fold changes of the respective genes in each sample [3]. The relative expression of all 11 CRM genes and the pathology was entered into Eureqa 1.24.0 (Nutonian, Boston, MA). Eureqa attempts to design a mathematical model that fits observed data employing an evolutionary algorithm. Symbolic regression was used to determine a relationship between the 11 genes and transplant phenotype. Models using 2 of the 11 CRM genes, consisting of ISG20 and CXCL9, were identified for tissue and BAL gene expression and used to calculate a modified 2-gene CRM score.

### Data analyses

All results are presented as median (interquartile range) or as mean ± SEM. Contingency tables were used to assess differences between groups with discrete data (Fisher exact or Chi-square test). Survival analysis was performed using Kaplan-M eier statistics. For continuous data, significances amongst groups were tested using the Student Mann–Whitney U test for two groups and the Kruskal-Wallis one-way analysis of variance in combination with Dunn’s post hoc test for multiple groups. For the CRM data, all p-values were adjusted using the Holm-Bonferroni method to correct for multiple testing. GraphPad Prism 6.0 software (San Diego, CA, USA) and R 3.2.2 statistical software (R Foundation for Statistical Computing, Vienna, Austria) was used for statistical analysis and a p-value <0.05 was considered statistically significant.

## Results

### Patient characteristics

TBB samples were obtained from 14 LTx recipients at 6 months post-LTx. Demographics of these patients are shown in Table 1. Of the 14 patients, 4 were confirmed to have acute rejection by LTx biopsy, while 10 did not show any evidence of rejection.

**Table 1:** Patient characteristics TBB samples.

	no AR	AR	p-value
**N**	10	4	
**Age, Y**	43 (36.25–49.50)	32.50 (23.75–48.75)	0.43
**Gender (M), N (%)**	5 (50)	2 (50)	> 0.05
**Indication for LTx, N (%)**			0.88
CF	1 (25)	1 (10)	
Emphysema	1 (25)	3 (30)	
ILD	1 (25)	4 (40)	
Other	1 (25)	2 (20)	
**Grade of acute rejection**	NA		
A1		0 (0)	
A2		0 (0)	
A3		4 (100)	
**brush cell amount, ng/μL**	196.70 (89.1–284)	209.60 (159.50–234.50)	> 0.05
**RNA yield, ng**	2556 (1158–3692)	2725 (2074–3049)	> 0.05
**260/280**	2.04 (1.94–3.08)	2.06 (2.04–2.09)	0.24

Abbreviations: LTx, lung transplantation; AR, acute rejection; CF, cystic fibrosis; ILD, interstitial lung disease. Results are shown in as median (IQR) or as numbers (percentage). P-values are displayed on the right and show the results of the Mann–Whitney test in case of continuous data. In case of discrete data, the results of the contingency table are shown. A p-value <0.05 was considered significant and is indicated in bold.

BAL samples at time of CLAD diagnosis were available from 10 RAS patients and 9 BOS patients whose tissue was included in this study. Additionally, BAL samples from 8 patients with AR were tested and 13 stable patients were used as controls. Demographics of these patients are shown in Table 2. At the time of sampling, FEV_1_ was significantly lower in BOS patients compared to stable patients (p = 0.028). No differences were present in FVC, FEV_1_/FVC ratio, and TLC between groups.

**Table 2:** Patient characteristics BAL samples.

	Stable	AR	BOS	RAS	p-value
**N**	13	8	9	10	
**Age, Y**	43 (34–55.50)	43.50 (21.50–54)	42 (23.50–58)	48.5 (24.75–53.25)	0.86
**Gender (M), N (%)**	4 (31)	4 (50)	4 (44)	7 (70)	0.32
**Indication for primary transplant, N (%)**					0.52
CF	5 (38.50)	3 (37.50)	3 (33)	1 (10)	
Emphysema	4 (31)	3 (37.50)	4 (44)	6 (60)	
ILD	2 (15.25)	2 (25)	1 (11.50)	0 (0)	
Other	2 (15.25)	0 (0)	1 (11.50)	3 (30)	
**Grade of acute rejection**	NA		NA	NA	
A1		0 (0)			
A2		6 (75)			
A3		2 (25)			
**Time between LTx and CLAD, Y**	NA	NA	4.82 (2.42–7)	4.63 (2.29–7.36)	0.79
**Time between CLAD and re-LTx, Y**	NA	NA	2.58 (0.94–2.97)	0.53 (0.23–2.18)	0.14
**FEV**_**1**_ **at BAL sampling, L**	2.69 (2–3.57)	2.19 (1.80–2.57)	1.60 (1.26–2.26)*	1.80 (1.45–2.92)	**0.034**
**FVC at BAL sampling, L**	3.37 (2.39–4.27)	2.53 (2.01–3.16)	2.94 (2.11–3.54)	2.72 (1.71–3.52)	0.45
**FEV**_**1**_**/FVC at BAL sampling, L**	0.83 (0.80–0.87)	0.89 (0.67–0.95)	0.47 (0.41–0.90)	0.70 (0.59–0.95)	0.19
**TLC at BAL sampling, L**	4.50 (3.47–5.95)	5.19 (4.13–6.08)	5.57 (4.77–6.37)	4.29 (3.92–5.55)	0.41
**mean best FEV**_**1**_**, L**	3.04 (2.16–4)	2.88 (2.21–3.55)	2.88 (2.05–3.14)	3.48(3.03–3.98)	0.19
**mean best FVC, L**	3.65 (2.65–4.69)	3.96 (2.58–4.17)	3.65 (3.09–4.13)	4.18 (3.75–5.69)	0.15
**mean best TLC, L**	5.02 (3.63–4.69)	5.19 (4.03–6.06)	5.99 (4.84–6.28)	6.15 (4.78–7.21)	0.35
**Cell profile**					
% Neutrophils	1.80 (1.10–3.10)	9.25 (6.38–16)	10.80 (1.70–29.30)	10.10 (1.70–23.85)	**0.024**
% Eosinophils	0 (0–0.10)	1 (0.13–1.75)	0.20 (0–0.50)	0.30 (0–2.38)	**0.046**
% Macrophages	93.20 (89.40–96)	80.75 (78.63–81.88)	86.60 (53.60–90.90)	74.20 (45.20–82)**	**0.002**
% Lymphocytes	2.60 (1.80–5.90)	9 (3.38–11.88)	8.40 (4.70–11)	8.20 (3.70–21.15)	0.17
**RNA yield, ng**	162 (49–337)	220 (42–950)	416 (46–916)	328 (196–3208)	0.24
**260/280**	1.66 (1.51–2.06)	1.86 (1.51–1.97)	1.94 (1.67–2)	1.90 (1.51–2.06)	0.75

Abbreviations: LTx, lung transplantation; AR, acute rejection; BOS, bronchiolitis obliterans syndrome; RAS, restrictive allograft syndrome; CLAD, chronic lung allograft dysfunction; CF, cystic fibrosis; ILD, interstitial lung disease; FEV_1_, forced expiratory volume in 1 s; FVC, forced vital capacity; TLC, total lung capacity; NA, not applicable. Results are shown in as median (IQR) or as numbers (percentage). P-values are displayed on the right and show the results of the Kruskal–Wallis ANOVA or Mann–Whitney test in case of continuous data. In case of discrete data, the results of the contingency table are shown. A p-value <0.05 was considered significant and is indicated in bold.

* p<0.05

** p<0.01 compared to stable patients

TBB and BAL samples were not obtained from the same patients. However, AR were all moderate to severe, depending on availability of samples. The grade of acute rejection tended to be higher in the TBB group (Tables 1 and 2, p = 0.06).

Samples obtained from explanted lungs at re-transplantation or autopsy (i.e. end-stage tissue) were available from 16 patients with RAS, 13 with BOS and 15 unused donor lungs. Demographics of these patients are shown in Table 3. There were fewer males with BOS compared to RAS and unused donor lungs (p = 0.03). At last contact, FEV_1_/FVC ratio was higher (p<0.0001) and TLC was lower (p = 0.03) in RAS patients compared to BOS patients, confirming the initial RAS diagnosis. RAS patients also had higher FEV_1_ values at last contact (p = 0.0029) and higher post-operative mean best FVC values (p = 0.03), compared to BOS patients. Survival post-CLAD diagnosis was significantly shorter in RAS patients (1.16 years) compared to BOS (2.63 years, p = 0.008).

**Table 3:** Patient characteristics explanted tissue.

	Control donor lungs	BOS	RAS	p-value
**N**	15	13	16	
**Age, Y**	52 (36.75–61.50)	46 (34–57.50)	45 (30–59)	0.47
**Gender (M), N (%)**	12 (80)	5 (38.50)	10 (63)	**0.03**
**Indication for primary transplant, N (%)**	NA			0.58
CF		5 (38.50)	4 (25)	
Emphysema		3 (23)	6 (37.50)	
ILD		4 (31)	3 (18.75)	
Other		1 (7.50)	3 (18.75)	
**Time between LTx and CLAD, Y**	NA	2.60 (1.30–4.80)	3.60 (2–6.70)	0.17
**Time between CLAD and re-LTx, Y**	NA	2.63 (1.43–4.72)	1.16 (0.52–2.04)	**0.008**
**FEV**_**1**_ **at last contact, L**	NA	0.60 (0.52–0.74)	0.88 (0.65–1.14)	**0.003**
**FVC at last contact, L**	NA	1.45 (1.28–2.14)	1.37 (1.15–1.58)	0.19
**FEV**_**1**_**/FVC at last contact, L**	NA	0.40 (0.29–0.43)	0.66 (0.54–0.78)	**<0.0001**
**TLC at last contact, L**	NA	5.41 (4.37–6.41)	4.28 (3.45–5.18)	**0.03**
**mean best FEV**_**1**_**, L**	NA	2.65 (1.83–3.23)	3.33 (2.74–3.85)	0.05
**mean best FVC, L**	NA	3.47 (2.74–4.15)	4.19 (3.70–5.27)	**0.03**
**mean best TLC, L**	NA	5.91 (4.15–6.32)	6.33 (4.78–7.21)	0.46
**RNA yield, ng**	5826 (4412–10142)	5288 (2327–8997)	7104 (3223–11672)	0.47
**260/280**	4.98 (1.96–2.02)	1.99 (1.95–2.02)	2 (1.96–2.03)	0.82

Abbreviations: LTx, lung transplantation; BOS, bronchiolitis obliterans syndrome; RAS, restrictive allograft syndrome; CLAD, chronic lung allograft dysfunction; CF, cystic fibrosis; ILD, interstitial lung disease; FEV_1_, forced expiratory volume in 1 s; FVC, forced vital capacity; TLC, total lung capacity; NA, not applicable. Results are shown in as median (IQR) or as numbers (percentage). P-values are displayed on the right and show the results of the Kruskal–Wallis ANOVA or Mann–Whitney test in case of continuous data. In case of discrete data, the results of the contingency table are shown. A p-value <0.05 was considered significant and is indicated in bold.

### CRM gene expression

**Fig 1:**
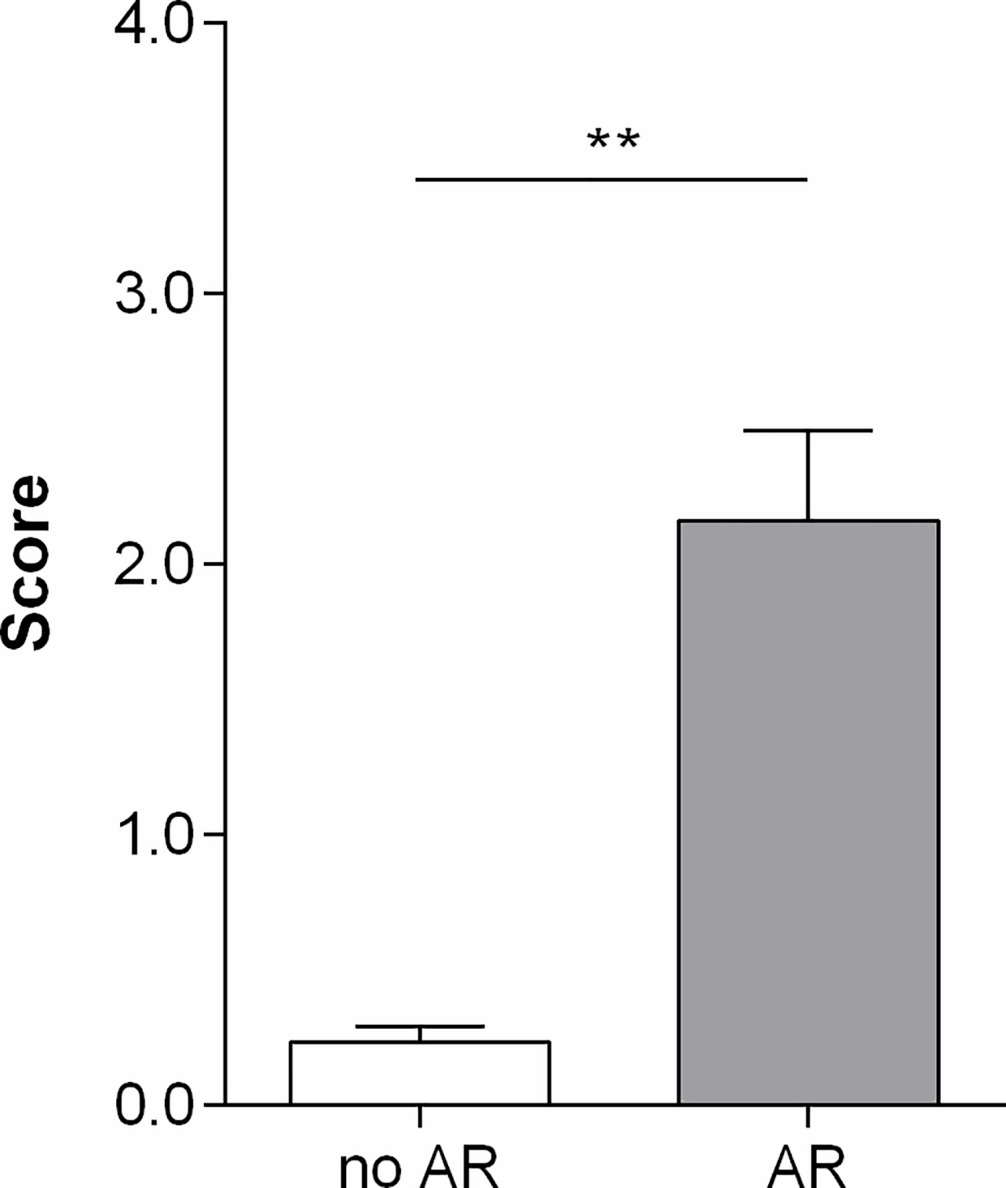
CRM score in TBB samples. Bar graph depicting the CRM score, based on a 2-gene model, in TBB samples of LTx recipients with AR (n = 4) and without AR (n = 10). Values are shown as mean ± SEM. Analysis was done using the Mann–Whitney test. A p-value <0.05 was considered significant. ** p<0.01.

In TBB samples comparing no AR to AR, there were significant differences in gene expression for all of the tested genes, except for BASP1 (TAP1, CXCL9, ISG20 and NKG7: p = 0.004, adjusted p = 0.032; CXCL10, INPP5D and RUNX3: p = 0.002, adjusted p = 0.026; LCK and PSMB9: p = 0.008, adjusted p = 0.032; BASP: p = 0.73; Table 4). However, the differential expression of CD6 lost significance after correction for multiple testing (p = 0.036, adjusted p = 0.072).The geometric means and CRM scores were lowest in the no AR group (1.69 and 0.17 respectively, Fig 1) compared to the AR group (6.61 and 2.19 respectively). The differences were significant for both scores (p = 0.002).

**Table 4:** TBB gene expression.

	no AR	AR	p-value
**CD6**	0.71	2.73	**0.036**
**TAP1**	1.84	9.49	**0.004 (0.032)**
**CXCL9**	1.44	34.81	**0.004 (0.032)**
**CXCL10**	1.24	17.28	**0.002 (0.026)**
**INPP5D**	0.8	1.92	**0.002 (0.026)**
**ISG20**	5.94	17.34	**0.004 (0.032)**
**LCK**	2.51	5.19	**0.008 (0.032)**
**NKG7**	0.46	2.56	**0.004 (0.032)**
**PSMB9**	3.92	13.37	**0.008 (0.032)**
**RUNX3**	1.06	2.83	**0.002 (0.026)**
**BASP**	3.84	6.61	0.73
***Geometric mean***	*1*.*69 (1*.*35–1*.*95)*	*6*.*61 (4*.*26–7*.*04)*	***0*.*002***
***CRM score***	*0*.*17 (0*.*12–0*.*30)*	*2*.*19 (1*.*51–2*.*79)*	***0*.*002***

Gene expression (fold change of Ct values) of the 11 CRM genes in TBB samples of LTx recipients with AR (n = 4) and without AR (n = 10). Values are presented as median. The geometric mean of expression of the individual CRM genes was calculated for each sample/patient and a CRM score was calculated based on a 2-gene model created in Eureqa. The median (IQR) of these scores is shown for each group at the bottom of the table. P-values are displayed on the right and show the results of the Mann–Whitney test. A p-value <0.05 was considered significant and is indicated in bold. Correction for multiple testing was performed using the Holm-Bonferroni method. When significant, adjusted p-values were added between brackets. Abbreviations: CD6, cluster of differentiation 6; TAP1, transporter associated with antigen processing 1; CXCL9, chemokine C-X-C motif ligand 9; CXCL10, chemokine C-X-C motif ligand 10; INPP5D, inositol polyphosphate-5-phosphatase D; ISG20, interferon-stimulated gene 20; LCK, lymphocyte-specific protein tyrosine kinase; NKG7, natural killer cell granule protein 7; PSMB9, proteasome subunit beta type-9; RUNX3, runt-related transcription factor 3; BASP1, brain abundant membrane attached signal protein 1; CRM, common rejection module.

In BAL samples comparing stable to AR LTx recipients, no difference in gene expression (CD6, TAP1, CXCL9, CXCL10, INPP5D, ISG20, LCK, NKG7, PSMB9, RUNX3 and BASP1) was detected (Table 5). The geometric mean (5.15 and 5.81, p = 0.50) and CRM score (0.72 and 0.56, p = 0.83) did not differ between stable and AR patients.

**Table 5:** AR BAL gene expression.

	Stable	AR	p-value
**CD6**	2.2	5.89	0.089
**TAP1**	0.72	1.03	0.69
**CXCL9**	9.66	10.57	0.89
**CXCL10**	5.25	8.95	0.99
**INPP5D**	6.93	7.2	0.96
**ISG20**	1.88	2.88	0.10
**LCK**	8.25	12.92	0.31
**NKG7**	1.72	4.87	0.30
**PSMB9**	15.96	22.67	0.50
**RUNX3**	5.51	9.96	0.37
**BASP**	9.41	8.24	0.91
***Geometric Mean***	*5*.*15 (3*.*08–10*.*17)*	*5*.*81 (3*.*48–22*.*07)*	*0*.*50*
***CRM Score***	*0*.*72 (0*.*46–0*.*88)*	*0*.*56 (0*.*08–18*.*31)*	*0*.*83*

Gene expression (fold change of Ct values) of the 11 CRM genes are shown in the form of a heat map for BAL samples of LTx recipients with AR (n = 8) and of stable patients (n = 13) at post-operative day 720 without any evidence of disease, used as controls. Values are presented as median. The geometric mean of expression of the individual CRM genes was calculated for each sample/patient and a CRM score was calculated based on a 2-gene model created in Eureqa. The median (IQR) of these scores is shown for each group at the bottom of the table. P-values are displayed on the right and show the results of the Mann–Whitney test. A p-value <0.05 was considered significant. After correction for multiple testing, all p-values were non-significant (data not shown). Abbreviations: CD6, cluster of differentiation 6; TAP1, transporter associated with antigen processing 1; CXCL9, chemokine C-X-C motif ligand 9; CXCL10, chemokine C-X-C motif ligand 10; INPP5D, inositol polyphosphate-5-phosphatase D; ISG20, interferon-stimulated gene 20; LCK, lymphocyte-specific protein tyrosine kinase; NKG7, natural killer cell granule protein 7; PSMB9, proteasome subunit beta type-9; RUNX3, runt-related transcription factor 3; BASP1, brain abundant membrane attached signal protein 1; CRM, common rejection module.

In BAL samples comparing stable, BOS and RAS patients, no difference in gene expression (CD6, TAP1, CXCL9, CXCL10, INPP5D, ISG20, LCK, NKG7, PSMB9, RUNX3 and BASP1) was detected. The geometric mean was the lowest for stable patients (5.15, Table 6), somewhat higher for BOS (5.62) and the highest for RAS patients (7.31). However, the differences between the various groups were not significant (p = 0.35). The CRM score, on the other hand, demonstrated significant differences between stable patients (CRM = 0.72) and BOS (0.98, p = 0.0008), and between stable patients and RAS (0.96, p = 0.013). No difference was demonstrated between CRM of BOS and RAS (p>0.05).

**Table 6:** BAL gene expression.

	Stable	BOS	RAS	p-value
**CD6**	2.20	4.74	9.10	0.11
**TAP1**	0.72	1.18	1.67	0.75
**CXCL9**	9.66	3.02	6.80	0.16
**CXCL10**	5.25	5.20	9.53	0.74
**INPP5D**	6.93	3.09	5.44	0.054
**ISG20**	1.88	3.76	2.71	0.54
**LCK**	8.25	6.63	10.47	0.29
**NKG7**	1.72	2.96	5.38	0.29
**PSMB9**	15.96	11.25	16.46	0.095
**RUNX3**	5.51	3.79	12.92	0.18
**BASP**	9.41	5.32	11.58	0.24
***Geometric Mean***	*5*.*15 (3*.*08–10*.*17)*	*5*.*62 (1*.*61–8*.*26)*	*7*.*31 (3*.*04–18*.*81)*	*0*.*35*
***CRM Score***	*0*.*72 (0*.*46–0*.*88)*	*0*.*98 (0*.*96–1*.27)***	*0*.*96 (0*.*83–1*.68)*	***0*.*0004***

Gene expression (fold change of Ct values) of the 11 CRM genes are shown in the form of a heat map for BAL samples of BOS (n = 9) and RAS (n = 10) patients. Samples of stable patients (n = 13) at post-operative day 720 without any evidence of disease were used as controls. Values are presented as median. The geometric mean of expression of the individual CRM genes was calculated for each sample/patient and a CRM score was calculated based on a 2-gene model created in Eureqa. The median (IQR) of these scores is shown for each group at the bottom of the table. P-values are displayed on the right and show the results of Kruskal-Wallis one-way ANOVA in combination with Dunn’s post hoc test. A p-value <0.05 was considered significant and is indicated in bold. After correction for multiple testing, all p-values were non-significant (data not shown).

* p<0.05

** p<0.01

*** p<0.001 compared to unused donor lungs.

Abbreviations: CD6, cluster of differentiation 6; TAP1, transporter associated with antigen processing 1; CXCL9, chemokine C-X-C motif ligand 9; CXCL10, chemokine C-X-C motif ligand 10; INPP5D, inositol polyphosphate-5-phosphatase D; ISG20, interferon-stimulated gene 20; LCK, lymphocyte-specific protein tyrosine kinase; NKG7, natural killer cell granule protein 7; PSMB9, proteasome subunit beta type-9; RUNX3, runt-related transcription factor 3; BASP1, brain abundant membrane attached signal protein 1; CRM, common rejection module.

**Fig 2:**
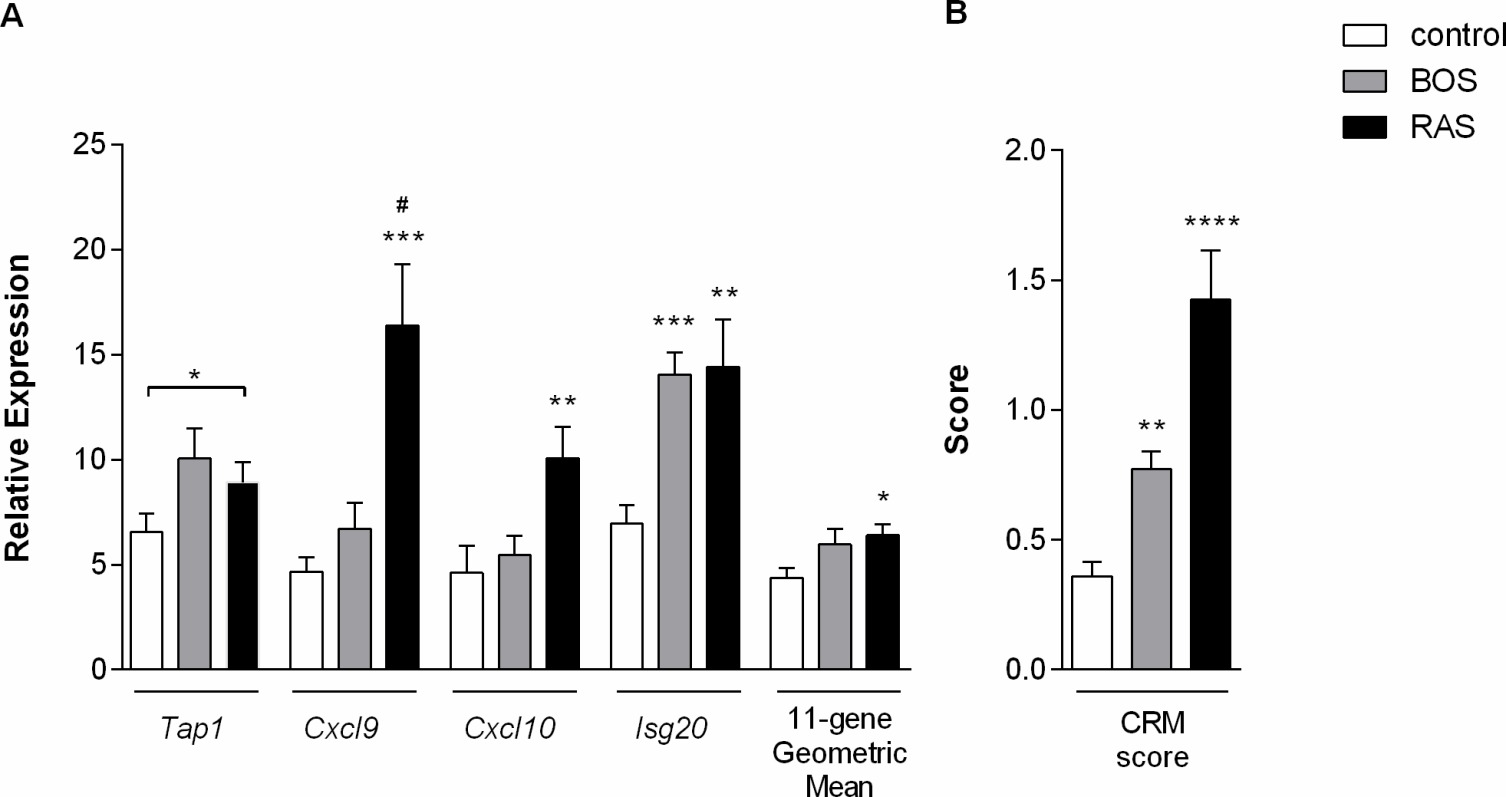
Differentially expressed genes in end-stage tissue. Bar graphs depicting (A) the expression of differentially expressed genes (TAP1, CXCL9, CXCL10, ISG20) and the geometric mean score and (B) the CRM score, based on a 2-gene model, in end-stage tissue of BOS (n = 13), RAS (n = 16) and unused donor lungs (control, n = 15). Values are shown as mean ± SEM. Analysis was done using Kruskal-Wallis one-way ANOVA in combination with Dunn’s post hoc test, not corrected for multiple testing. A p-value <0.05 was considered significant. * p<0.05, ** p<0.01, *** p<0.001, **** p≤0.0001 compared to unused donor lungs; # p<0.05 compared to BOS lungs.

In explant tissue, there was a significant difference in expression of TAP1 (p = 0.041), CXCL9 (p = 0.0007), CXCL10 (p = 0.009) and ISG20 (p = 0.0004; Table 7 and Fig 2). RAS lungs showed higher expression of CXCL10 (p = 0.0079) and ISG20 (p = 0.0081) compared to unused donor lungs, while CXCL9 was upregulated in RAS compared to both unused donor lungs (p = 0.0006) and BOS lungs (p = 0.039). The differential expression of TAP1 could not be attributed to a specific difference within groups. In BOS lungs, only ISG20 gene expression was upregulated compared to unused donor lungs (p = 0.0007). After correction for multiple testing, the differential expression of TAP1 (adjusted p = 0.33) and CXCL10 (adjusted p = 0.09) lost significance. There was no difference in expression of the other genes measured in this assay (CD6, INPP5D, LCK, NKG7, PSMB9, RUNX3 and BASP1).

**Table 7:** Tissue gene expression.

	Control donor lungs	BOS	RAS	p-value
**CD6**	3.82	4.25	7.09	0.16
**TAP1**	**6.07**	**8.34**	**9.54**	**0.041**
**CXCL9**	**4.18**	**5.77**	13.48***^,^^**#**^	**0.0007 (0.0077)**
**CXCL10**	**3.68**	**5.57**	8.1**	**0.009**
**INPP5D**	3.97	3.63	3.64	0.83
**ISG20**	**6.59**	12.62^***^	12.19**	**0.0004 (0.0048)**
**LCK**	5.16	5.57	6.89	0.40
**NKG7**	4.62	5.19	4.96	0.51
**PSMB9**	6.06	7.92	6.75	0.34
**RUNX3**	5.45	7.69	7.9	0.079
**BASP**	0.74	1.02	0.96	0.25
***Geometric Mean***	*4*.*09 (3*.*22–5*.*06)*	*5*.*37 (4*.*68–6*.*33)*	*6*.*35 (4*.*70–7*.75)*	***0*.*018***
***CRM Score***	*0*.*35 (0*.*15–0*.*43)*	*0*.*69 (0*.*54–1*.02)**	*1*.*49 (0*.*61–2*.11)****	***<0*.*0001***

Gene expression (fold change of Ct values) of the 11 CRM genes in end-stage tissue of BOS (n = 13), RAS (n = 16) and control donor lungs (n = 15) are shown in the form of a heat map. Values are presented as median. The geometric mean of expression of the individual CRM genes was calculated for each sample/patient and a CRM score was calculated based on a 2-gene model created in Eureqa. The median (IQR) of these scores is shown for each group at the bottom of the table. P-values are displayed on the right and show the results of Kruskal-Wallis one-way ANOVA in combination with Dunn’s post hoc test. A p-value < 0.05 was considered significant and is indicated in bold. Correction for multiple testing was performed using the Holm-Bonferroni method. When significant, adjusted p-values were added between brackets.

* p<0.05

** p<0.01

*** p<0.001

**** p≤0.0001 compared to unused donor lungs

^#^ p<0.05 compared to BOS lungs.

Abbreviations: CD6, cluster of differentiation 6; TAP1, transporter associated with antigen processing 1; CXCL9, chemokine C-X-C motif ligand 9; CXCL10, chemokine C-X-C motif ligand 10; INPP5D, inositol polyphosphate-5-phosphatase D; ISG20, interferon-stimulated gene 20; LCK, lymphocyte-specific protein tyrosine kinase; NKG7, natural killer cell granule protein 7; PSMB9, proteasome subunit beta type-9; RUNX3, runt-related transcription factor 3; BASP, brain abundant membrane attached signal protein; CRM, common rejection module.

The geometric mean of expression of the individual CRM genes was calculated for each sample/patient, as described previously [3] (Table 7). The geometric mean was the lowest in unused donor tissue (4.09), somewhat higher in BOS (5.37) and the highest in RAS (6.35), with a significant difference between RAS and unused donor lungs (p = 0.018). The CRM score, defined by a 2-gene model designed by Eureqa, demonstrated significant differences between unused donor tissue (CRM = 0.35) and BOS (0.69, p = 0.0067) or RAS (1.49, p<0.0001), but not between BOS and RAS (p>0.05). However, a receiver-operating characteristic (ROC) curve analysis was used to evaluate the 2-gene model of the CRM score and demonstrated that the model is able to distinguish RAS from BOS in explanted tissue (AUC = 0.75, 95% CI = 0.56–0.94, p = 0.020). This was not the case in BAL samples (data not shown).

Within RAS tissue, different parenchymal patterns were observed (organizing pneumonia, n = 9 or 56.25%; acute fibrinous organizing pneumonia, n = 4 or 25%; pleuroparenchymal fibro-elastosis, n = 7 or 43.75%). There were no differences in geometric mean or CRM score when comparing the different patterns (data not shown), however, power was low for this sub analysis.

There was no correlation between gene expression in BAL and in tissue within samples derived from the same patients. Furthermore, there was no correlation between geometric means or CRM scores and total or differential cell count in BAL (data not shown).

## Discussion

Non-invasive and reliable monitoring of both acute and chronic rejection is indispensable in transplant medicine. Nevertheless, there are currently few–if any–biomarkers to monitor and predict allograft rejection. The development of a system to assess messenger RNA levels in kidney transplant biopsies has enabled the diagnosis of specific disease phenotypes within renal failure and improved risk stratification [16,17]. Moreover, the system has recently been adapted to assess heart transplant specimens [18]. The recent identification of a CRM score for rejection in microarray datasets from different types of solid tissue allografts (kidney, heart, liver, lung) and its further validation in renal allografts encouraged the idea that increased risk of allograft-injury could be predicted, maybe even before the actual damage has occurred in LTx patients [3,4,19]. A computational score across all 11 genes in kidney transplantation also predicted which patients would more likely develop chronic rejection over time in the same study.

In this study, we utilized BAL samples and independent LTx tissue samples to evaluate the predictive capacity of the CRM gene-set to detect acute and chronic LTx injury by carefully selecting tissue samples from LTx patients with acute rejection and well-characterized diagnosis of CLAD, segregated into BOS and RAS. Instead of using microarrays, as in the discovery study, the 11 CRM genes were evaluated by multiplex quantitative PCR. Moreover, comparing the expression of the same gene-set in BAL samples from the same patients at CLAD diagnosis allowed us to assess if these genes can be used for non-invasive diagnosis of LTx chronic injury, which was based on the prior observation (Sarwal *et al*, in submission) that the CRM gene-set mirrors the same injury profile in kidney transplant tissue and paired urine samples.

BAL presents itself as the preferable tool to assess the micro-environment of the lung allograft–and to possibly detect CLAD–as LTx recipients undergo bronchoscopy as part of routine follow-up in most hospitals. In a recent review, Kennedy and colleagues ranked the strength of associations between BAL parameters and BOS, showing that neutrophil count, interleukin (IL)-8, alpha defensins and matrix metalloproteinase 9 demonstrated highly replicable associations with BOS [20]. The current different CLAD phenotypes were, however, not considered in these published studies.

Our results from TBB samples from patients with and without AR showed differential gene expression of 10 of the 11 CRM genes. This is consistent with our findings in the original CRM paper, where the 11 CRM genes were identified in transplant acute rejection inclusive of lung tissue. That the majority of genes were significantly upregulated compared to the analyses of the BOS and RAS tissue samples suggests that while there is some degree of transcriptional overlap between acute and chronic rejection, these are distinct processes. Hypotheses regarding the relationship between acute and chronic rejection have suggested both a temporal and immunological association [19]. It is possible that chronic rejection in the lung is reflective of ongoing, low-grade inflammation that insidiously leads to a fibrotic state as compared to the high-grade inflammation seen with acute rejection.

In explanted LTx tissue, RAS samples showed significant changes in the expression of a subset of genes from the CRM module, specifically TAP1, CXCL9, CXCL10 and ISG20, resulting in a significant elevation of the geometric mean score, compared to unused donor lungs. A CRM score created based on ISG20 and CXCL9, was able to significantly discriminate RAS and BOS from unused donor lungs. Our results showed differential gene expression interferon-stimulated gene 20 (ISG20) in both BOS and RAS, while expression of CXCL9 (also referred to as monokine induced by gamma interferon, MIG) and CXCL10 (also referred to as interferon gamma-induced protein 10, IP-10) was only upregulated in RAS tissue. These results partially correspond to a previous study of our research group, where we investigated BAL protein expression in BOS and RAS [21], showing that CXCL10/IP-10, but also IL-1β, IL-1Rα, IL-6, IL-8, MCP-1/CCL2, MIP-1α/CCL3, MIP-1β/CCL4, and VEGF were upregulated in RAS. CXCL9/MIG protein levels were, however, not different between CLAD phenotypes. The chemokines CXCL9 and CXCL10 are interferon-γ induced CXCR3 ligands that act as chemo-attractants for monocytes and lymphocytes, such as natural killer cells and activated T-cells. In addition, Shino and colleagues implicated this CXCR3/ligand axis in the association between diffuse alveolar damage and increased risk of CLAD [22] and recently demonstrated that elevated CXCR3 ligand levels in BAL significantly increases the risk of CLAD development–more specifically RAS–during organizing pneumonia. Moreover, they suggest the potential use of CXCR3 ligands in BAL fluid as a prognostic biomarker after lung transplantation [23]. The biological activities and specific mechanisms of action of interferon-stimulated exonuclease gene 20, or ISG20, remain unclear. However, its involvement in antiviral mechanisms has been demonstrated [24] and it is suggested to play a role in certain inflammatory responses, such as rheumatoid arthritis and multiple sclerosis and could be an interesting biomarker [25].

It is remarkable that, both in acute and chronic settings, we could demonstrate a difference in gene expression in TBB and in end-stage explant tissue but not in lavage samples. This suggests that BAL might not be an appropriate monitoring tool; however, it could also be explained by the adopted BAL procedure, in which 2x50 cc of saline is used, which probably only samples the larger airways. Bollman and colleagues recently stressed the importance of instillation protocols, demonstrated that a 5x20 lavage yielded higher BAL recovery and lower neutrophil and lymphocyte percentages than a 2x50 cc lavage [26]. In an endeavour to limit BAL result variability, the International Society for Heart and Lung Transplantation has currently set up a committee to assess and formulate recommendations for BAL standardization. It is also important to note that investigating protein expression in BAL samples is more compartment specific–only looking in the airways–while investigating gene expression in tissue specimens includes several compartments.

It is of interest that the gene expression signature in RAS tissue shows more similarities to rejection post-kidney/liver/heart transplantation than BOS. Given that interstitial fibrosis is a common feature across organ rejection and is typical for RAS we wonder if RAS should be seen as the ‘true’ form of chronic lung allograft rejection, which leaves us questioning what BOS exactly represents. This question, however, needs further investigation.

We acknowledge that this study has some drawbacks. First, the study was limited by the number of available samples as well as the restricted panel of genes/proteins investigated. Examining more genes/proteins would allow us to form a more complete picture of possible underlying mechanisms and potential biomarkers. Moreover, RNA extraction of sufficient quality from BAL samples proved difficult, leading to lower 260/280 ratios than commonly used. However, a 260/280 ratio of at least 1.4 was reached and several research groups have discussed the use of inferior quality RNA in gene expression analysis, demonstrating that this did not affect amplification of target genes [13–15]. A plus point in this study is the use of samples from a well-characterized and -phenotyped group of CLAD patients. Moreover, both BAL and tissue samples were available from the same patients, providing us the unique possibility to compare results of the two compartments. Furthermore, gathering many samples remains difficult due to rarity of the disease and the low incidence of (re-) transplantations/autopsies.

In summary, we assessed the CRM in acute and chronic rejection post-lung transplantation, with special consideration for the different CLAD phenotypes. The CRM score in tissue was able to distinguish RAS from BOS and donor control lungs. Consequently, we believe that similar underlying mechanisms may be in place in RAS and rejection after kidney/liver/heart transplantation. Analysis of BAL samples showed no differential gene expression in either acute or chronic settings; therefore, tissue might be a more appropriate tool for predicting and/or diagnosing CLAD.
